# Recombinant Salivary Proteins of *Phlebotomus orientalis* are Suitable Antigens to Measure Exposure of Domestic Animals to Sand Fly Bites

**DOI:** 10.1371/journal.pntd.0004553

**Published:** 2016-03-17

**Authors:** Michal Sima, Blanka Ferencova, Alon Warburg, Iva Rohousova, Petr Volf

**Affiliations:** 1 Department of Parasitology, Faculty of Science, Charles University in Prague, Prague, Czech Republic; 2 Department of Microbiology and Molecular Genetics, The Kuvin Centre for the Study of Infectious and Tropical Diseases, The Hebrew University - Hadassah Medical School, The Hebrew University of Jerusalem, Jerusalem, Israel; University of Notre Dame, UNITED STATES

## Abstract

**Background:**

Certain salivary proteins of phlebotomine sand flies injected into the host skin during blood-feeding are highly antigenic and elicit strong antibody-mediated immune responses in repeatedly-exposed hosts. These antibodies can be measured by enzyme-linked immuno sorbent assays (ELISAs) using salivary gland homogenates (SGHs) as the source of antigens and serve as a markers for exposure to biting sand flies. Large-scale screening for anti-sand fly saliva antibodies requires replacement of SGH with recombinant salivary proteins. In East Africa, *Phlebotomus orientalis* is the main vector of *Leishmania donovani*, a trypanosomatid parasite causing visceral leishmaniasis. We tested recombinant salivary proteins derived from *Ph*. *orientalis* saliva to study exposure of domestic animals to this sand fly species.

**Methodology/Principal Findings:**

Antigenic salivary proteins from *Ph*. *orientalis* were identified by immunoblot and mass spectrometry. Recombinant apyrase rPorSP15, yellow-related protein rPorSP24, ParSP25-like protein rPorSP65, D7-related protein rPorSP67, and antigen 5-related protein rPorSP76 were tested using ELISA with sera of domestic animals from *L*. *donovani* foci in Ethiopia where *Ph*. *orientalis* is present. Our results highlighted recombinant yellow-related protein rPorSP24 as the most promising antigen, displaying a high positive correlation coefficient as well as good sensitivity and specificity when compared to SGH. This recombinant protein was the most suitable one for testing sera of dogs, sheep, and goats. In addition, a different antigen, rPorSP65 was found efficacious for testing canine sera.

**Conclusions/Significance:**

Recombinant salivary proteins of *Ph*. *orientalis*, specifically rPorSP24, were shown to successfully substitute SGH in serological experiments to measure exposure of domestic animals to *Ph*. *orientalis*, the vector of *L*. *donovani*. The results suggest that rPorSP24 might be a suitable antigen for detecting anti-*Ph*. *orientalis* antibody-mediated reactions also in other host species.

## Author Summary

The sand fly *Phlebotomus orientalis* is the main vector of *Leishmania donovani*, the causative agent of visceral leishmaniasis in East Africa. During bloodfeeding, sand flies inject saliva into the host skin and repeated bites result in a specific antibody response in the bitten hosts. Antibody responses are directed against sand fly salivary proteins and the levels of these antibodies reflect the intensity of exposure to biting sand flies. The antibody reactions can be measured using salivary gland homogenates (SGHs), but for large-scale testing its use is impractical because of the amount of work required to obtain sufficient quantities of SGH. Recombinant proteins prepared based on the antigens in the sand fly saliva can substitute whole SGH in large-scale studies. We tested five recombinant proteins from *Ph*. *orientalis* saliva expressed in *Escherichia coli* and demonstrated that the yellow-related protein rPorSP24 can replace the SGH in estimating exposure to sand flies of dogs, goats, and sheep in Ethiopia. Immune reactions to vector saliva in endemic areas, provides useful information on levels of exposure and, thereby, on the effectiveness of vector control programs.

## Introduction

Phlebotomine sand flies are the vectors of *Leishmania* parasites causing leishmaniasis, the disease responsible for an estimated 1.3 million new human cases and 20 000 to 30 000 deaths annually [1]. During blood-feeding, sand fly females inoculate saliva into the host skin. Over the last three decades, various research groups have investigated the composition and biological activities of saliva, as well the potential use of salivary antigens in an anti-*Leishmania* vaccine (reviewed in [2]).

Sand fly salivary molecules are also highly antigenic and elicit strong antibody-mediated response in repeatedly exposed hosts. This response can be utilized as a marker for exposure to biting sand flies. In animals experimentally-exposed to sand fly bites the production of specific anti-saliva IgG antibodies is positively correlated with the number of blood-fed sand flies [3,4]. The elevated antibody levels persisted in bitten hosts for weeks or even months [3–6] but decreased after the last exposure to sand flies, suggesting that screening for anti-saliva antibodies can be used also for estimating the timing of exposure [7,8]. As a reliable epidemiological tool, anti-sand fly saliva antibodies have already been successfully employed to evaluate the effectiveness of vector control interventions [4,9], to estimate the risk of *Leishmania* transmission [4, 10–12], and to indicate the feeding preferences of sand flies [13–15].

Screening for anti-sand fly saliva antibodies in large populations is impractical due to the amount of work required to obtain sufficient quantities of salivary gland homogenate (SGH). However, the use of recombinant salivary proteins enables to circumvent the necessity for sand fly colony maintenance, laborious dissections of salivary glands and potential cross-reactivity with non-vector species [8]. The main salivary antigens in several sand fly species have already been characterized [4,12,16–18], however, recombinant salivary proteins from only three species—*Lutzomyia longipalpis*, *Ph*. *perniciosus*, and *Ph*. *papatasi*—have been tested so far in seroepidemiological studies [13,19–25].

Here, we focus on *Ph*. *orientalis*, the most important vector of human visceral leishmaniasis (VL) in East Africa [reviewed in [26]]. In Ethiopia, the main endemic areas of VL are located in the lowlands of southwestern Ethiopia and in the Metema-Humera plains in the northwest [27], where *Ph*. *orientalis* was found to be an abundant sand fly species [28]. This opportunistic sand fly feeds on different mammals, depending on the host availability [29–31]. Indeed, anti-*Ph*. *orientalis* antibodies have recently been detected in several domestic animal species in Ethiopia—dogs, donkeys, sheep, goats, and cows using SGH as antigen [15]. In the present study, five proteins from saliva of *Ph*. *orientalis* were expressed in *Esherichia coli* and evaluated as markers for exposure using sera of domestic animals, namely dogs, sheep, and goats, from *L*. *donovani* endemic foci in northern Ethiopia.

## Methods

### Ethical statement

BALB/c mice were maintained and handled in the animal facility of Charles University in Prague in accordance with institutional guidelines and the Czech legislation (Act No. 246/ 1992 coll. on Protection of Animals against Cruelty in present statutes at large), which complies with all relevant European Union and international guidelines for experimental animals. The experiments were approved by the Committee on the Ethics of Animal Experiments of the Charles University in Prague (Permit Number: 24773/2008-10001) and were performed under the Certificate of Competency (Registration Number: CZ 02439, CZ 02457) in accordance with the Examination Order approved by Central Commission for Animal Welfare of the Czech Republic. Sera of domestic animals were collected within the study by [15]. Their collection was approved by the Ethiopian National Research Ethics Review Committee (NRERC) under approval no. 3.10/3398/04. For more details see [15].

### Host sera

Murine sera were obtained from animals exposed at least ten-times to about 150 insectary-bred sand fly females of a single species in two-week interval. Ten mice were exposed *to Ph*. *orientalis*, four to *Ph*. *papatasi*, and four to *Sergentomyia schwetzi*. Four mice served as non-exposed controls. Serum samples of Ethiopian domestic animals, obtained during the previous study by [15] included 179 sheep, 36 dog, and 233 goat sera. Sera from 30 sheep, 14 dogs, and 15 goats non-exposed to sand flies were used as negative controls. More details about samples from domestic animals (both of Ethiopian origin and controls) are provided in [15].

### Sand flies and salivary gland dissection

The colony of *Ph*. *orientalis* (originating from Ethiopia, Melka Werer) was established in 2008 [32] and reared under standard conditions as described in [33]. Salivary glands were dissected from 4–6 day old female sand flies in 20 mM Tris buffer with 150 mM NaCl and stored at -20°C. Before use, salivary glands were disrupted by freeze-thawing three times in liquid nitrogen [34].

### Immunoblot

Antigenic proteins were selected based on the reactivity of two pools of canine sera (five sera each) from endemic area in Ethiopia with SGH using one pool (five sera) of non-exposed dogs as control. Salivary proteins (equivalent to 20 glands per well) were separated by SDS-PAGE on 12% polyacrylamide gel under non-reducing conditions. Proteins were transferred from the gel to nitrocellulose membranes using an iBLOT dry system (Invitrogen). Membranes were cut into strips and blocked overnight with 5% nonfat dry milk in Tris buffer with 0.05% Tween (Tris-Tw) and incubated for 1 hour with dog sera diluted 1:50 in Tris-Tw. Then, the strips were incubated with peroxidase conjugated anti-dog IgG (Bethyl Laboratories) diluted 1:3000 in Tris-Tw. Antigenic protein bands were visualized using the substrate solution with diaminobenzidine. The protein profile was compared with *Ph*. *orientalis* SGH studied by [35] and the identity of antigenic bands was confirmed by proteome analysis and mass spectrometry; according to the protocol described in [35].

### Recombinant proteins preparation

Five proteins from *Ph*. *orientalis* salivary glands were chosen for expression in *E*. *coli*: PorSP15, PorSP24, PorSP65, PorSP67, and PorSP76 (in [35] marked as PorASP15, PorMSP24, PorMSP65, and PorMSP67, and PorASP76, respectively) (Table 1). The PCR products from a previously constructed cDNA library [35] were used as the starting material. Products were amplified by PCR under the following conditions: initial incubation (3 minutes in 94°C), then 30 cycles of denaturation (30 seconds in 94°C), annealing (1 minute in 57°C), and elongation (1 minute in 72°C). The whole reaction was terminated by heating to 72°C for 10 minutes. Specific primers were synthesized according to the sequences of the mature protein (without the signal peptide) (Table 1). Thereafter, we followed the procedure described in [5]. Briefly, PCR products were ligated into *E*. *coli* pGEM-T Easy Vector (Promega) using TA cloning and the ligation products were transfected into *E*. *coli* competent cells TOP10 (Invitrogen). Vectors were replicated in bacteria and after that, the gene for yellow-related protein was restricted using Spe I and Xho I and the genes for the remaining four proteins were restricted using Nde I and Xho I enzymes. Restricted *E*. *coli* pET-42 Expression Vectors (Novagen) were ligated and ligation products were transformed into *E*. *coli* competent cells TOP10 (Invitrogen) again. Plasmids were isolated from the bacteria, and transfected into *E*. *coli* BL21 (DE3) gold (Agilent) for expression. *E*. *coli* lysates were prepared under denaturing conditions and His-tagged proteins (with six histidins) were purified under denaturing conditions with 8M urea in Ni-NTA column (731–1550: Bio-Rad, USA). Purity of the recombinant proteins was verified on immunoblot using the monoclonal anti-polyHistidine-peroxidase (A7058-1VL: Sigma Aldrich, UK) and protein concentration was measured by the Lowry method (Bio-Rad) following the manufacturer’s protocol.

**Table 1:** *Phlebotomus orientalis* salivary proteins expressed in *Escherichia coli*.

Sequence name	Protein family	GenBank ACCN	Forward primer	Reverse primer
**PorSP15**	Apyrase	AGT96431	CATATGGCTCCTAGAGCAACAAAAT	CTCGAGCTTAATGCCTTTGGGAT
**PorSP24**	Yellow-related protein	AGT96428	ACTAGTTTTCACGTTGAAAGAGAAT	CTCGAGCTTTGTCTTGGGATCATA
**PorSP65**	ParSP25-like protein	AGT96466	CATATGGATCGGGGAGTGGATGG	CTCGAGGTGCAATCGGTTGTTTATG
**PorSP67**	D7-related protein	AGT96467	CATATGCTGCAATTCCCTCGTGAT	CTCGAGTTTTGCCGATATCTCATCC
**PorSP76**	Antigen 5-related protein	AGT96441	CATATGGCTAACGACTATTGCCAGC	CTCGAGTGTCCTGGGCTTCTTGAG

Sequence name, the protein family, GenBank accession number, and forward and reverse primers for PCR amplification are provided for each protein.

### ELISA experiments

The ELISA protocol described in [15] was used with the following modification: The ELISA plates Immulon 4HBX (96w flat bottomed plate, 735–0465: VWR, USA) were coated in concentrations of 5 μg/ml (0.5 μg/well) for recombinant proteins and 28 ng/well for SGH (corresponding to 0.2 of salivary gland/well). In all ELISA tests, serum samples were tested individually.

In the first series of experiments (evaluation step), sera from ten experimentally-bitten mice and sera of 10 dogs, 10 goats, and 35 sheep with the highest anti-*Ph*. *orientalis* SGH titer values found by [15] were used to evaluate the antigenicity of the five recombinant proteins with anti-*Ph*. *orientalis* saliva IgG. Sera from non-exposed animals (3 mouse, 3 dogs, 3 goats, and 8 sheep) were used as negative controls.

In the second series of experiments, 16 murine sera (4 exposed to *Ph*. *orientalis*, 4 to *Ph*. *papatasi*, 4 to *Se*. *schwetzi*, and 4 non-exposed controls) were used to verify specificity of selected recombinant proteins. Based on the results of evaluation experiments with murine sera, three recombinant proteins with significant correlation with SGH (rPorSP24, rPorSP67, and rPorSP76) were selected.

In the third series of experiments (validation step), selected recombinant proteins with correlation coefficient higher than 0.7 from evaluation experiments (rPorSP15, rPorSP24, rPorSP65, and rPorSP67) were tested with the whole set of serum samples from Ethiopia (179 sheep, 36 dogs, and 233 goats) and an appropriate number of non-exposed controls (30 sheep, 14 dogs, and 15 goats).

### Statistical analysis

The non-parametric Spearman test was used to assess correlations between total anti-SGH and anti-recombinant protein IgG levels using GraphPad Prism version 6 (GraphPad Software, Inc., San Diego, CA). For evaluating the possible cross-reactivity with other sand fly species non-parametric Wilcoxon Rank-Sum test in GraphPad Prism version 6 (GraphPad Software, Inc., San Diego, CA) was used. Statistical significance was considered when the p-value was<0.05. Cut-off values were calculated from the mean optical density of control sera plus 3 standard deviations. The optical density values of anti-SGH antibodies were used as the gold standard to validate recombinant proteins in ELISA tests using positive and negative predictive values, sensitivity, and specificity.

### Accesion numbers

Accession numbers of proteins used in this study: AGT96431, AGT96428, AGT96466, AGT96467, AGT96441. Accession numbers of proteins discussed in this study: AAL16051, AHA49643, AAL11049, AAL11048, AFY13224, ABI20147, AHF48995, AHF48996, AAD32198, AAS05318, AHF49000

## Results

### Identification of *Ph*. *orientalis* salivary antigens by immunoblot

**Fig 1:**
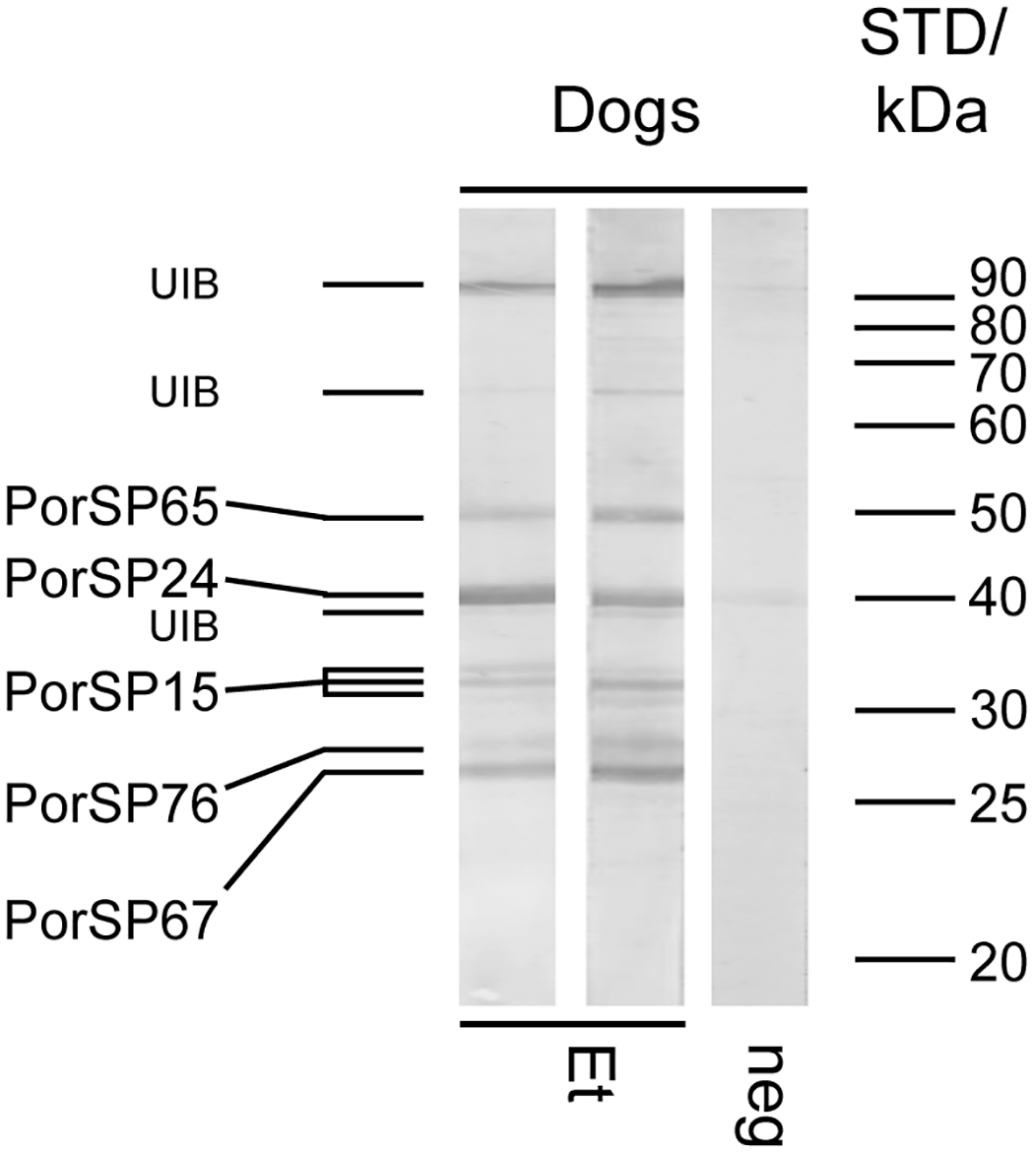
Identification of *Phlebotomus orientalis* salivary antigens in dogs. *Ph*. *orientalis* salivary proteins were separated under non-reducing conditions by SDS-PAGE on a 12% gel and incubated with two different pools of sera from naturally-exposed Ethiopian dogs (Et), and one pooled sera from non-exposed control dogs (neg). Each pool was a mixture of 5 individual samples. Five antigenic proteins (PorSP65, PorSP24, PorSP15, PorSP76, and PorSP67) were identified by successive proteome analysis and mass spectrometry. Molecular weights of standard (STD) are indicated. UIB means unidentified bands.

To identify antigenic proteins in *Ph*. *orientalis* salivary glands, two pools of canine sera (five sera each) from naturally-exposed dogs were tested with SGH of *Ph*. *orientalis*. Individual bands were identified based on the proteomic analysis, immunoblot, and mass spectrometry of *Ph*. *orientalis* SGH. Canine sera reacted with at least 10 protein bands (Fig 1); five of them were identified as ParSP25-like protein PorSP65, yellow-related protein PorSP24, apyrase PorSP15, antigen 5-related protein PorSP76, and D7-related protein PorSP67 (for GenBank ACCN refer to Table 1). These five antigenic proteins were chosen for expression in *E*. *coli*. Very weak reaction was observed between SGH and negative control sera around 30, 40, and 90 kDa (Fig 1).

### Evaluation of recombinant proteins with anti-*Ph*. *orientalis* IgG

To evaluate the reactivity of anti-*Ph*. *orientalis* saliva IgG with five recombinant proteins, we screened them first with selected sera of naturally-exposed domestic animals from Ethiopia (dogs, sheep, and goats), mice experimentally-bitten by *Ph*. *orientalis*, and non-exposed controls. The antigenicity of recombinant proteins was evaluated based on the correlation of antibody reactions with SGH for each of the twenty combinations between recombinant proteins and animal species (Table 2).

**Table 2:** Evaluation of recombinant proteins by correlation analysis.

HOST		SGH	rPorSP15	rPorSP24	rPorSP65	rPorSP67	rPorSP76
**Dog**	controls (n = 3)	0.396 ± 0.049	**0.329 ± 0.037**	**0.289 ± 0.008**	**0.278 ± 0.038**	0.341± 0.050	0.332 ± 0.053
	exposed (n = 10)	1.129 ± 0.336	**0.709 ± 0.181**	**0.734 ± 0.232**	**1.008 ± 0.304**	0.742 ± 0.227	0.755 ± 0.230
	ρ	N.A.	0.830 ***	0.868 ***	0.852 ***	0.687 **	0.599 *
**Goat**	Controls (n = 3)	0.143 ± 0.002	0.215 ± 0.021	**0.150 ± 0.023**	0.161 ± 0.030	**0.213 ± 0.031**	0.354 ± 0.058
	exposed (n = 10)	0.265 ± 0.224	0.559 ± 0.244	**0.329 ± 0.234**	0.411 ± 0.234	**0.422 ± 0.202**	0.737 ± 0.230
	ρ	N.A.	0.797 ***	0.835 ***	0.412	0.802 ***	0.643*
**Sheep**	controls (n = 8)	0.133 ± 0.014	0.294 ± 0.041	**0.213 ± 0.048**	0.166 ± 0.027	0.288 ± 0.047	0.520 ± 0.113
	exposed (n = 35)	0.218 ± 0.141	0.486 ± 0.138	**0.238 ± 0.165**	0.313 ± 0.180	0.353 ± 0.084	0.573 ± 0.091
	ρ	N.A.	0.629 ***	0.806 ***	0.777 ***	0.373 *	0.349 *
**Mouse**	controls (n = 3)	0.086 ± 0.006	0.158 ± 0.022	**0.142 ± 0.037**	0.154 ± 0.034	0.136 ± 0.029	**0.214 ± 0.059**
	exposed (n = 10)	1.395 ± 0.562	0.245 ± 0.186	**0.394 ± 0.329**	0.753 ± 0.761	0.334 ± 0.314	**0.391 ± 0.136**
	ρ	N.A.	0.308	0.857 ***	0.456	0.560 *	0.802 ***

Spearman-Rank Correlation Matrix test for optical densities between sera tested against *Ph*. *orientalis* SGH and against each salivary recombinant protein. Mean values of exposed animals and non-exposed controls ± standard deviation, correlation coefficient (ρ) are indicated. N.A. = not applicable, asterisk (*) indicates significant correlations: *p<0.05, **p<0.01, ***p<0.001. Combinations of significant correlation and ρ>0.8 are in bold.

In canine sera, a significant correlation was achieved for all tested proteins; the highest correlation coefficient was found for rPorSP24 (ρ = 0.868), followed by rPorSP65, and rPorSP15. Similarly, in sheep and goat sera the best correlation (above 0.8) was found for rPorSP24, other recombinant proteins with correlation coefficient above 0.75 were rPorSP67 and rPorSP15 for goats and rPorSP65 for sheep (Table 2). Sera from experimentally-bitten mice showed significant correlation with three out of five proteins tested; the highest correlation coefficient was achieved for rPorSP24 (ρ = 0.857).

### Specificity of recombinant proteins

**Fig 2:**
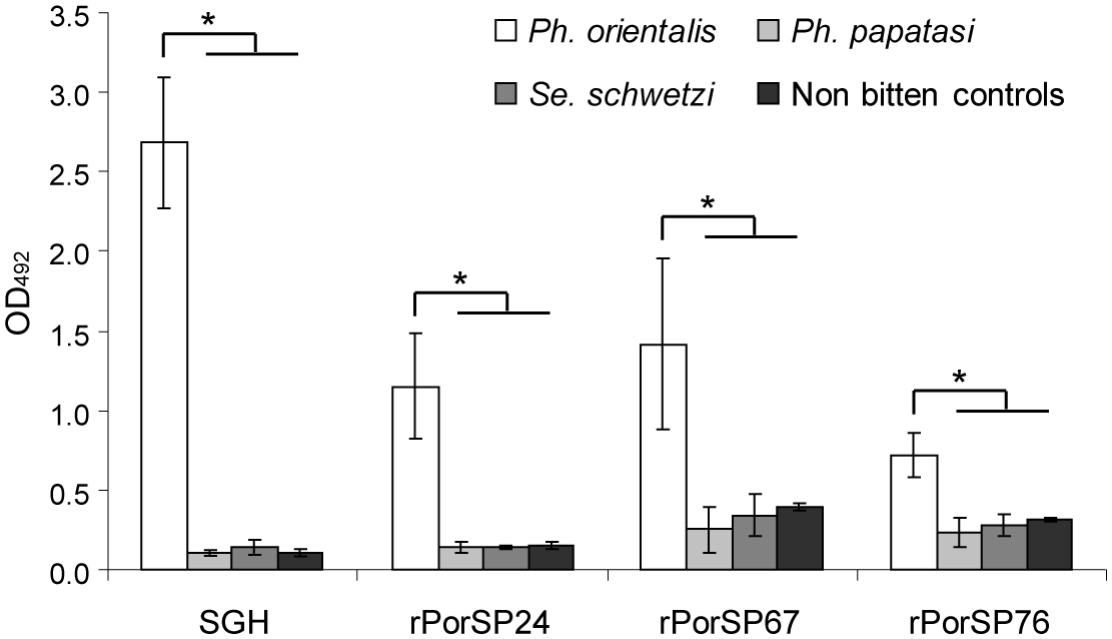
Specificity of recombinant proteins. Results from ELISA are presented as mean optical densities ± standard deviation of IgG antibody reaction with *P*. *orientalis* salivary gland homogenate (SGH) and three recombinant proteins (rPorSP24, rPorSP67, and rPorSP76) in mice experimentally bitten by *Ph*. *orientalis*, *Ph*. *papatasi*, or *Se*. *schwetzi*, and non-exposed control mice. Four mice per sand fly colony and four non-exposed controls were used. Asterisks (*) indicate significant differences (p<0.05, calculated with non-parametric Wilcoxon Rank-Sum Test) of IgG levels between mice bitten by *Ph*. *orientalis* and mice bitten by other sand fly species or non-bitten controls.

Specificity of recombinant proteins was tested only with murine sera due to the absence of positive control samples from other host species. Three recombinant proteins with significant correlation to SGH in evaluation experiments with murine sera (Table 2) were selected to verify their specific reaction with anti-*Ph*. *orientalis* IgG. Fig 2 shows the strong reactions of sera from mice exposed to *Ph*. *orientalis* bites with SGH and with all tested recombinant proteins (rPorSP67, rPorSP76, and rPorSP24), while the recognition of these antigens by sera from mice exposed to solely to *Ph*. *papatasi* or *Se*. *schwetzi* were similar to the negative controls (sera of unexposed mice).

### Validation of selected recombinant proteins

Four recombinant proteins with the highest correlation from evaluation experiments (rPorSP15, rPorSP24 rPorSP65, and rPorSP67) were chosen for further validation using the whole set of Ethiopian serum samples (179 sheep, 36 dogs, and 233 goats) and non-exposed controls.

**Fig 3:**
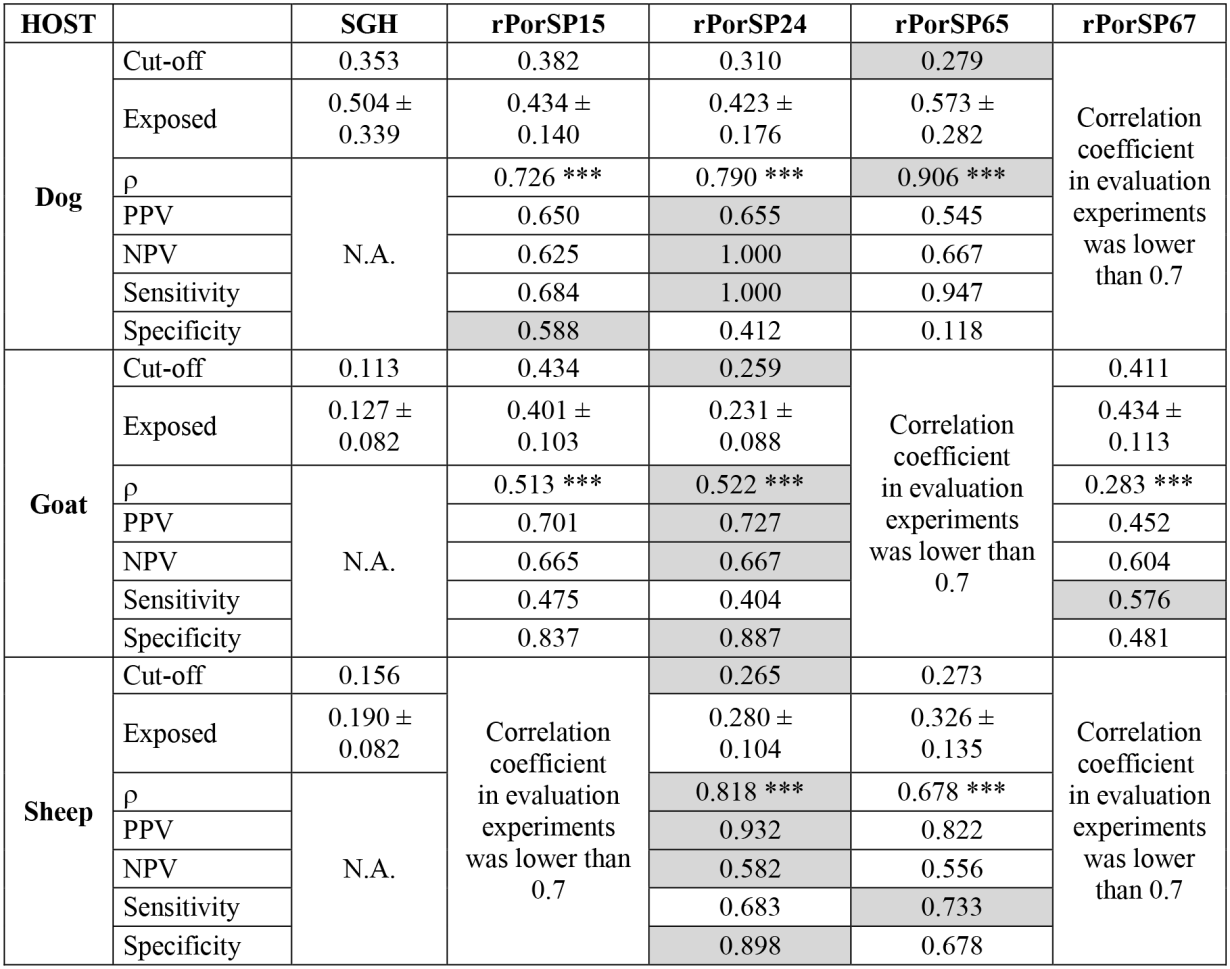
Validation of recombinant proteins. Selected recombinant proteins were validated in ELISA tests with sera of indicated domestic animals naturally-exposed to *Phlebotomus orientalis*. The analysis was based on comparison with anti-*Ph*. *orientalis* SGH IgG as a standard. The table provides cut-off values, mean values of optical density ± standard deviation of IgG levels in animals exposed to *Ph*. *orientalis*, correlation coefficients between IgG levels against SGH and a recombinant protein (ρ), positive predictive values (PPV), negative predictive values (NPV), sensitivity, and specificity. Asterisks (*) indicate significant correlations: *p<0.05, **p<0.01, ***p<0.001. Combinations with the correlation coefficient lower than 0.7 in evaluation experiments (Table 2) were excluded from the validation experiments. For each combination, the lowest cut-off value, the highest correlation coefficient, and the highest PPV, NPV, sensitivity, and specificity values are shaded grey. N.A. means not applicable.

**Fig 4:**
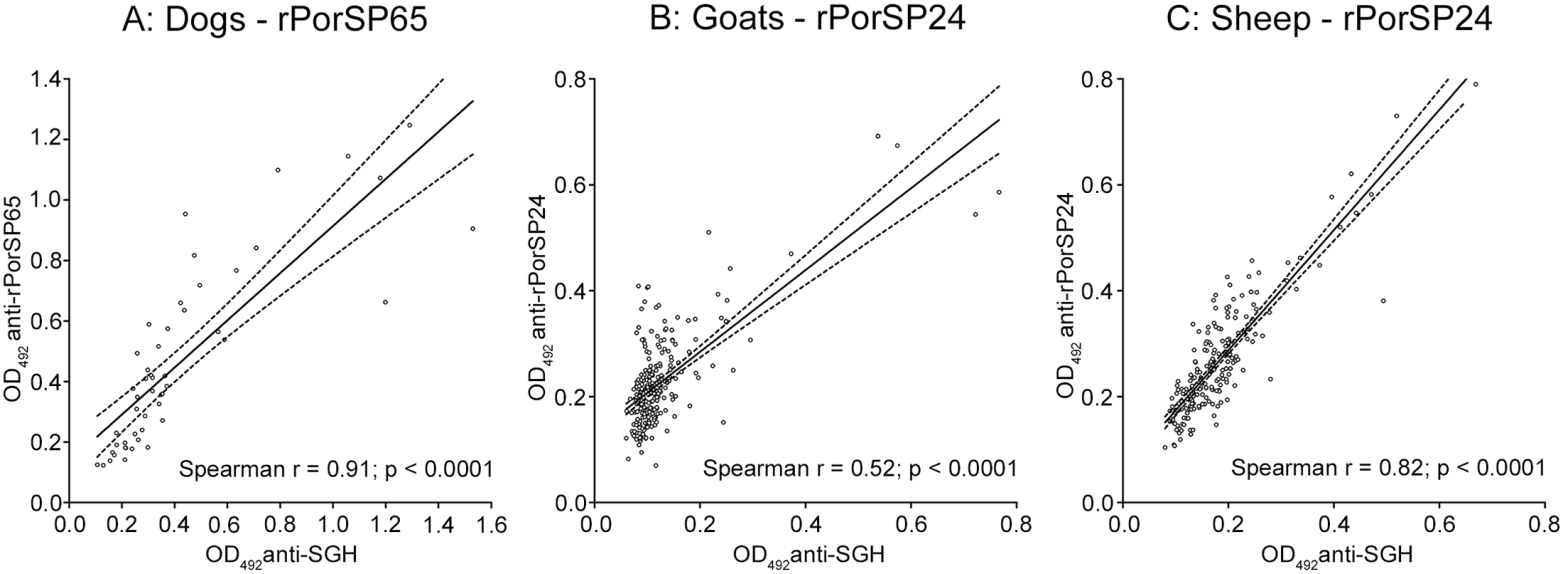
Correlation analyzes between IgG antibodies against SGH and recombinants rPorSP65 and rPorSP24 in ELISA. For each animal species from validation experiments (Fig 3), the protein with the highest positive correlation was displayed: A: rPorSP65 (ParSP25-like protein) tested with canine sera (n = 50), B: rPorSP24 (yellow-related protein) tested with goat sera (n = 248), C: rPorSP24 (yellow-related protein) tested with sheep sera (n = 209). Sera from naturally-exposed Ethiopian animals together with sera from non-exposed controls were included in this analysis. Correlation coefficients and p-values from Spearman-Rank analysis are indicated.

For canine sera, the highest correlation coefficient was achieved with rPorSP65 (ρ = 0.906) followed by rPorSP24 and PorSP15. For sheep as well as for goats, the highest correlation coefficient was detected with rPorSP24 (ρ = 0.818 and ρ = 0.522, respectively) followed with rPorSP65 for sheep and with rPorSP15 and rPorSP67 for goats (Fig 3). All results from correlation analyses between SGH and four recombinant proteins were highly significant and cut-off values for individual recombinant proteins were the lowest for the proteins with the highest correlation coefficient (Fig 3). Additionally, rPorSP24 reached the highest values of positive and negative predictive values (PPV and NPV) in all host species as well as the sensitivity in dogs and the specificity in goats and sheep. The specificity in dogs was the best with rPorSP15 and the sensitivity in goats with rPorSP67. The best combinations (the lowest cut-off value, the highest correlation coefficient, PPV, NPV, specificity, and sensitivity values) between SGH and recombinant protein for each animal species tested are shown in Fig 4.

## Discussion

We have studied antigenic salivary proteins of *Ph*. *orientalis*, the most important vector of VL in Ethiopia and Sudan, using sera of naturally-exposed hosts. Antigenic proteins were identified on immunoblot based on their recognition by canine sera. These were: D7-related protein (PorSP67), antigen 5-related protein (PorSP76), apyrase (PorSP15), yellow-related protein (PorSP24), and ParSP25-like protein (PorSP65). The antigenicity of their recombinant counterparts expressed in *E*. *coli* was validated in large-scale tests using sera from naturally-exposed dogs, sheep, and goats. The utilization of recombinant proteins as markers for exposure may help to highlight the specificity of the reaction and to evade nonspecific binding as observed when SGH was recognized by negative control. sera.

Sand fly salivary proteins from the D7-related family are well known antigens; they were recognized by sera of mice bitten by *Ph*. *papatasi* [10], dogs bitten by *Lu*. *longipalpis* and *Ph*. *perniciosus* [4,36], and humans bitten by *Ph*. *papatasi* [12]. As far as we are aware, five recombinant D7-related (rD7) proteins from sand fly saliva have already been tested as exposure markers; one from *Lu*. *longipalpis* (AAL16051, also known as LJL13) [13], another from *Ph*. *perniciosus* (AHA49643) [21], and three from *Ph*. *papatasi* (AAL11049, AAL11048, and AFY13224) [5,20]. However, only some of them bound anti-saliva IgG and their antigenicity was host-specific. The rD7 protein (AAL11049) was specifically recognized by sera from mice bitten by *Ph*. *papatasi* [5], but the same protein was not recognized by human sera from Tunisia [20]. Another rD7 protein (AAL11048) from *Ph*. *papatasi* did not react with sera from mice immunized by sand flies [5]. The rD7 protein LJL13 was recognized by dogs naturally-exposed to *Lu*. *longipalpis* but not by sera from foxes and humans from endemic focus of *L*. *infantum* [13]. In the present study, rPorSP67 showed promising results with limited number of goat sera during the evaluation test but was not validated in a broader test, when medium or low correlation coefficient, NPV, PPV, sensitivity, and specificity with values ranging between 0.28 and 0.6 were observed. This suggests that this recombinant protein would not be useful as an exposure marker to *Ph*. *orientalis* bites.

Proteins of the antigen 5-related family from various sand fly species were repeatedly shown to be potent salivary antigens, being recognized by sera of mice bitten by *Ph*. *papatasi* and *Ph*. *arabicus* [5,16], dogs bitten by *Ph*. *perniciosus* [4], rabbits exposed to *Ph*. *tobbi* [18], and hamsters bitten by *Ph*. *argentipes* [37]. In our study, rPorSP76 showed high correlation with SGH only with sera from experimentally-bitten mice (ρ = 0.8), thus its use in field studies with domestic animals is not justified.

Apyrases are well-known salivary enzymes with anti-haemostatic properties [38]. Antigenic properties of apyrases were described in SGHs of various vector-host models such as *Ph*. *perniciosus* and dogs [4, 17] or *Ph*. *argentipes* and hamsters [37]. Recombinant apyrase (ABI20147) was recognised by sera of mice immunized with *Ph*. *duboscqi* saliva [39]. Two apyrases in a recombinant form (AHF48995, AHF48996) were used as exposure markers for dogs, hares, and rabbits bitten by *Ph*. *perniciosus* [21,22], however, the most recent work by Kostalova et al.[23] revealed that neither of these recombinant proteins gave optimal ELISA results in large-scale tests with naturally-exposed dogs. Our results showed a significant correlation between antibody response against SGH of *Ph*. *orientalis* and recombinant apyrase rPorSP15 in sera of all domestic animals tested but not in mice sera. The best correlation (ρ = 0.7) was observed with canine sera with medium values of NPV, PPV, sensitivity, and specificity (ranging between 0.59 and 0.68) suggesting necessity of further validation before its utilization as antigen for detecting dog exposure to *Ph*. *orientalis*.

The most promising and universal candidate for an exposure marker to sand fly bites belongs to the family of yellow-related proteins. Strong antibody responses to these proteins were previously demonstrated for various sand fly and host species, including dogs and humans [3–7,10,12–14,17,36,40]. *Ph*. *perniciosus* recombinant yellow-related protein AHF49000 was successfully used as an antigen both in ELISA and immunoblot reacting well with sera of mice and dogs experimentally-bitten by *Ph*. *perniciosus* [21]. Yellow-related recombinant proteins were also validated as exposure markers to sand fly bites in endemic areas; *Lu*. *longipalpis* AAD32198 and AAS05318 for humans [19,13] and *Ph*. *perniciosus* AHF49000 for dogs, rabbits, and hares [22,23]. In the present study, rPorSP24 from *Ph*. *orientalis* confirmed the high reactivity of yellow-related protein family with antibodies from sera of bitten hosts and this advocates for its use as an exposure marker in large-scale field studies. It reached high correlation with SGH in mice (ρ = 0.9), sheep (ρ = 0.8), and dogs (ρ = 0.8) and the best in goats (ρ = 0.5). Similarly, high correlation between SGH and recombinant yellow-related protein was previously attained with dogs (ρ > 0.7 [21–23]), hares (ρ = 0.9 [22]), and rabbits (ρ = 0.7 [22]). Moreover, rPorSP24 achieved the highest values of specificity, sensitivity, PPV and NPV with majority of the host species tested. In dogs, this recombinant protein showed 100% NPV and sensitivity but lower values of PPV and specificity (0.66 and 0.41, respectively), indicating higher probability of false positivity among non-exposed dogs. On the other hand, in sheep, very high values of PPV and specificity were achieved (both 0.9) but medium values of NPV and sensitivity (0.58 and 0.68, respectively) could indicate possible false negative results. However, this statistical analysis is based on data from naturally-exposed hosts and negative controls; further validation is needed using sera of experimentally-bitten animals as a positive control.

The fifth recombinant protein tested belongs to the ParSP25-like family. Antigenicity of this protein family was previously demonstrated in *Ph*. *perniciosus* [4,17]. So far as we are aware, no recombinant protein from this group was prepared and used for measuring the antibody reaction with sera of bitten hosts. Our results suggest significant correlation between rPorSP65 and antibody response against SGH of *Ph*. *orientalis*. The highest correlation coefficient was observed with canine sera (ρ = 0.9), accompanied by high degree of sensitivity (0.95). Nevertheless, the specificity of the test with rPorSP65 was very low (0.1) suggesting high probability of false positivity among non-exposed dogs.

Antigenic specificity of recombinant proteins was confirmed by using murine sera experimentally-exposed to *Sergentomyia schwetzi* or *Phlebotomus papatasi*. These two sand fly species are present in Ethiopia, in some places sympatrically with *Ph*. *orientalis* [27]. Antibodies from sera of mice bitten by *Ph*. *papatasi* or *Se*. *schwetzi* did not react with the recombinant proteins with significant correlation from evaluation experiments (rPorSP24, rPorSP67, and rPorSP76) and confirmed that they are species-specific.

The reactivity of recombinant proteins might be affected by the expression system or conditions of protein purification. Antibodies from bitten hosts can be targeted also to the glycosylated parts of the antigen that are lacking in proteins from the *E*. *coli* expression system, therefore some authors prefer to express recombinant proteins in mammalian cells [20,24]. Nevertheless, some of the recombinant proteins without posttranslational modifications proved to be as efficient markers of exposure as native antigens [5,21–23]. Similarly, several proteins prepared in this study were found as suitable antigens, despite their being expressed in *E*. *coli*.

In conclusion, our study suggests rPorSP24, the recombinant protein from the yellow-related family, as the most reliable and universally efficacious antigen for measuring exposure of dogs, sheep and goats to *Ph*. *orientalis* bites. In addition, the recombinant protein rPorSP65 from ParSP25-like group was found as a good antigen to screen for canine exposure but its low specificity suggests possible false positivity in some specimens. Serological tests using these proteins could be a highly practical and economical tool for screening of domestic animals for exposure to the main vector of *L*. *donovani* in East Africa. Moreover, the promising characteristics of rPorSP24 suggest a potential use of this antigen for screening sera of other hosts, including humans. The availability of recombinant salivary proteins should enable to measure anti-*Ph*. *orientalis* antibodies in large-scale experiments to evaluate vector control programs in areas affected by VL in East Africa. However, further studies are needed to validate such recombinant protein-based test for routine use.
